# Digital public health interventions for the promotion of mental well-being and health behaviors among university students: a rapid review

**DOI:** 10.1186/s12889-025-23669-1

**Published:** 2025-07-18

**Authors:** Paula M. Matos Fialho, Vanessa Wenig, Eileen Heumann, Maria Müller, Christiane Stock, Claudia R. Pischke

**Affiliations:** 1 Medical Faculty and University Hospital Duesseldorf, Centre for Health and Society, Institute of Medical Sociology, Heinrich Heine University Duesseldorf, Moorenstr. 5, Duesseldorf, Germany; 2 Institute of Health and Nursing Science, Charité– Universitätsmedizin Berlin, corporate member of Freie Universität Berlin and Humboldt-Universität zu Berlin, Berlin, Germany; 3 University of Southern Denmark, Unit for Health Promotion Research, Esbjerg, Denmark

**Keywords:** Digital public health interventions, Mental health, University students, Well-being, Health behavior

## Abstract

**Background:**

Student life offers many opportunities for personal development; however, this transitional phase often also poses challenges to mental health. Various factors associated with university life, such as academic stress and financial burdens, have been found to exacerbate psychological distress and contribute to increased alcohol and substance use among students. Our aim is to closely examine (i) components of available digital public health interventions (DPHIs), (ii) to evaluate their effectiveness for promoting well-being, health behaviors, and reducing symptoms of mental disorders among university students and (iii) to rate the quality of the evidence identified in this rapid review.

**Methods:**

We conducted a rapid review to capture the evidence on DPHIs for university students. We adhered to the methodological criteria recommended by the Cochrane Rapid Reviews Methods Group and PRISMA. The literature search was performed in Ovid MEDLINE to look for articles related to university students, mental well-being, mental health, and DPHIs published between June 2018 - June 2023. The selection was carried out in two steps: Screening of titles and abstracts and screening of full texts.

**Results:**

One thousand one hundred thirty-two articles were screened, of which 24 met the inclusion criteria for data extraction. (i) Of the intervention components in the included studies, 18 used web-based platforms, while six used smartphone applications. The interventions were aimed at improving mental health (68%), reducing substance use (28%), promoting physical activity (PA) (36%) and changing eating habits (16%). (ii) 42% of the included studies were able to demonstrate significant effects in the intended direction for mental health, 4% for substance use, 25% for PA and 8% for eating behavior. (iii) The quality assessment revealed that 58% of the studies were classified with ‘some concerns’, indicating moderate bias, while 29% were classified as ‘high risk’, suggesting considerable bias affecting the validity of the results.

**Conclusion:**

This rapid review comprehensively summarized intervention components of DPHIs. Further, the findings of this review provide significant insights into the effectiveness of DPHIs targeting mental well-being and health behaviors among university students. The studies included in the analysis demonstrate varying degrees of success across different domains, highlighting both the potential and limitations of DPHIs.

**Supplementary Information:**

The online version contains supplementary material available at 10.1186/s12889-025-23669-1.

## Introduction

University years generally represent a period of personal growth and development towards emerging adulthood [1]. However, due to leaving home, taking on financial responsibilities for the first time, and adjusting to a new social environment, it may also be a vulnerable time full of challenges and life changes. Thus, university students take on greater responsibility and explore their identity in a new and potentially unstable social environment [1, 2].

The pressure and changes associated with university life can have a negative impact on students’ mental health [2]: University students are susceptible to mental health issues, such as anxiety, stress, depression, as well as unfavorable substance use behavior [3–6]. The World Health Organization (WHO) documented that 35.3% of 13.984 first-year university students, from 19 universities across eight countries, scored positive for at least one prevalent Diagnostic and Statistical Manual of Mental Disorders IV (DSM-IV) anxiety, mood, or substance disorder [5]. Despite the availability of treatment, only one in six university students received minimally adequate treatment for mental disorders [5]. In addition to mental health challenges, university students often exhibit unhealthy lifestyle behaviors, such as physical inactivity and consumption of an insufficient amount of fruits and vegetables [7]. This transitional phase from late adolescence to adulthood is critical, as the adoption of such behaviors increases the risk of developing chronic non-communicable diseases, such as cardiovascular diseases, obesity, and type 2 diabetes [8, 9]. Furthermore, these unhealthy lifestyle choices are not only detrimental to physical health, but can also exacerbate mental health issues (e.g., increase the risk for depression [10]).

The recent Coronavirus Disease 2019 (COVID-19) pandemic exacerbated the challenges to mental health [11, 12]. During this time, university students may have experienced additional stress due to limited social interactions with peers, increased expectations for self-directed learning skills, and concerns about successfully completing their academic studies [13, 14]. Long-term adverse effects can impact on both individuals and society as a whole, as poor mental health may reduce academic performance, increase the likelihood of dropping out of college, and lead to impaired functioning in later stages of life [15].

Comprehensive interventions are essential for maintaining and improving the mental well-being of university students, given the high vulnerability and the previously reported, existing treatment gap [5, 15]. It is important to distinguish between mental health, which refers to a state of psychological functioning enabling individuals to cope with stress and contribute to society [16], and mental well-being, which emphasizes positive emotions, such as satisfaction and personal flourishing [17].

Given these widespread and often unmet mental health needs among university students, innovative and scalable solutions are required. One such approach involves the use of digital public health interventions (DPHIs), which use technology to promote mental well-being and facilitate behavior change [18, 19]. Considering the increase in digital literacy and internet use among young individuals, DPHIs may offer a growing opportunity to improve the accessibility of mental health interventions to university students [20, 21]. Students’ receptiveness to digital health applications, facilitated by time-saving attributes and widespread smartphone access, has been further accentuated by the familiarity gained during the COVID-19 pandemic, especially through online learning [22]. Recognized by the WHO, DPHIs share similarities with traditional public health interventions, holding the potential to address a broad spectrum of mental health problems [23]. The overall aim of DPHIs is to promote healthy lifestyles and general well-being [18, 23]. The different instruments of DPHIs, including websites, games, applications (apps), robots, virtual reality, and mobile text messaging, offer flexibility and technical diversity for usage across various mental health concerns. They have been recognized for their potential to reduce accessibility barriers, as being effective, accessible, anonymous, providing prompt feedback, applicable in real-life contexts, and maintaining high treatment fidelity [24, 25].

Previous research found that mobile-based apps, standalone self-help interventions, and blended concepts are effective in treating anxiety, depression, sleep problems, symptoms of stress, and alcohol use post-traumatic stress, and eating disorders among university students [22, 25, 26]. According to Liverpool et al. [20],, remote mental health outpatient care had a lower cancellation rate compared to face-to-face appointments. This is particularly important when considering the existing barriers to seeking help among university students. Concerns about stigma, confidentiality and feelings of shame or embarrassment about discussing personal issues are known to deter students from accessing traditional mental health services [27, 28]. DPHIs provide an alternative means of support for university students who may face barriers, potentially making support more accessible [20, 28].

In this review, we define mental health promotion as interventions that aim to improve psychological well-being and support positive mental health, regardless of the presence or absence of mental illness. Mental health promotion focuses on strengthening protective factors, fostering resilience, and building skills to manage stress and emotions in everyday life [29]. This is conceptually distinct from prevention strategies, which are typically categorized as primary (preventing the onset of mental illness), secondary (early identification and intervention), and tertiary (reducing the impact of established mental disorders) prevention [30]. While there may be some overlap between mental health promotion and primary prevention, our review specifically included interventions aimed at improving the psychological well-being and health behaviors of the general student population, rather than at diagnosing, treating, or managing existing mental health conditions.

Hence, using digital tools, DPHIs offer an expanded opportunity to effectively reach university students. Their affinity to digital communication ensures that DPHIs are easily accessible and provide mental health resources that fit seamlessly into young people’s digital lifestyles. Research on the effectiveness of DPHIs for university students is a growing field. Many publications each year focus on their potential to alleviate symptoms of mental health disorder and enhance psychological well-being. Building on the growing interest in digital health, several reviews have synthesized evidence on digital interventions aimed at providing psychological support to university students. For instance, Harith et al. [31] conducted an umbrella review highlighting the overall effectiveness of digital mental health interventions for reducing symptoms of depression, anxiety, and stress among university students. Montagni et al. [21] examined usage patterns and identified common barriers, such as concerns about confidentiality and perceived usefulness, which may hinder engagement. D’Adamo et al. [32] focused on cognitive-behavioral therapy (CBT)-based digital tools, reporting generally positive psychological outcomes, though with substantial variability in reach and user engagement. Bolinski et al. [33] examined the academic impact of e-mental health tools and found a small but statistically significant improvement in performance, while Dick et al. [34] reviewed harm reduction strategies for substance use, emphasizing the need for tailored, context-sensitive digital approaches.

While these reviews offer important contributions, they are often limited in scope—focusing narrowly on specific therapeutic models (e.g., CBT or ACT), a single outcome domain (e.g., mental health or academic performance), or a particular intervention format (e.g., mobile apps or online modules). Most have not concurrently examined broader health behaviors, such as physical activity, dietary habits, or substance use, nor have they systematically assessed structural characteristics, such as delivery format and level of intervention (individual vs. population-based). What is missing in the current literature is a comprehensive and integrative synthesis that captures the diversity and complexity of DPHIs targeting university students. Our rapid review addresses this gap by offering an up-to-date overview of DPHIs, analyzing not only mental well-being outcomes but also health-related behaviors. Further, we classify interventions by type (individual vs. multi-level), delivery mode, and core components; assess their effectiveness across behavioral domains; and critically appraise their methodological quality using a standardized risk of bias tool. Hence, the current review helps to bridge the existing gaps outlined above and offers guidance for the development and implementation of future digital interventions in higher education settings.

This review adopts a global perspective, including studies conducted in diverse geographical regions (rural/urban) without restricting inclusion by country. The population focus is on university students (without a restriction regarding age or gender), regardless of nationality reflecting the widespread relevance of DPHIs for this demographic group.

The following research questions are addressed:

i) Which digital interventions aimed at improving mental well-being and health behaviors in university students have been implemented and tested for effectiveness in the past five years, and which components and intervention levels were included in these interventions?
ii) How effective were these interventions for improving wellbeing overall, health behaviors, and reducing symptoms of mental illness?
iii) What was the quality of the evidence identified in this rapid review?

## Methods

This rapid review adheres to the Guidance for Rapid Reviews established by Cochrane and is reported in accordance with the Preferred Reporting Items for Systematic Reviews and Meta-Analyses (PRISMA) guidelines [35]. Compared to a systematic review, a rapid review is a streamlined form of evidence synthesis that uses abbreviated methods, such as a limited search scope or a simplified appraisal,- to produce timely and policy relevant findings, while still ensuring methodological transparency and rigor [36]. The protocol was registered at PROSPERO prior to screening (registration number: CRD42023442264).

### Search strategy

To enhance the methodological rigor of our study, our rapid review team strategically prioritized a bibliographic database to strike a balance between sensitivity and accuracy in the search process. After thorough evaluation, it was determined that OVID MEDLINE offers comprehensive coverage aligning closely with both the topic and design of our study. Consequently, we utilized this database as the primary source for our rapid review.

### Search criteria and selection

Eligible studies encompassed randomized controlled trials (RCTs) investigating the promotion of mental health and health behaviors among university students through digital interventions. Interventions were eligible irrespective of their content, duration, and setting. The intervention had to have been carried out digitally (i.e., web-based interventions accessed via computer, laptop, tablet, smartphone). Suitable studies were expected to evaluate at least one outcome related to mental well-being (i.e., symptoms relating to depression, anxiety, psychological distress, and stress) or health behavior (physical activity, substance use, including cannabis use, smoking, alcohol use, and eating behavior).

Eligible studies also had to be published in peer-reviewed journals and findings presented in English. The literature search was conducted in July 2023. The search strategy was designed to capture studies published over a 5-year period, specifically from June 2018 to June 2023. This period was chosen to provide a comprehensive overview of DPHIs before and after the onset of the COVID-19 pandemic, allowing us to compare interventions implemented in the two years before and after the pandemic. The complete list of search terms and search syntax can be found in Multimedia Appendix 1.

We applied exclusion criteria to ensure the review focused only on interventions for health promotion. Studies were excluded if the digital intervention was developed primarily to treat an existing diagnosed mental health condition.

Two independent reviewers (PMF and VW) screened the titles and abstracts of retrieved articles to identify potentially relevant studies in Rayyan [37]. Full-text articles meeting the predefined eligibility criteria were then obtained for further assessment. Screening of full-text articles was conducted by three independent reviewers (PMF, VW, EH). Interrater reliability was almost perfect at title/abstract level (kappa = 0.98) and full-text level (kappa = 0.94). At both stages of screening, disagreements were resolved through discussion between the reviewers.

### Data extraction

The data extraction sheet was specifically designed for this review to capture key information from each study, including study characteristics (e.g., authors, publication year, study design), participant demographics (e.g., age, gender), intervention details (e.g., type, duration), outcomes assessed (e.g., measures of mental well-being, health behavior), components (i.e., the type of digital intervention used, such as Internet-Based Cognitive Behavioral Therapy (CBT) or Acceptance and Commitment Therapy (ACT)) and results. Descriptive data from eligible studies were extracted by one reviewer (MM) and subsequently crosschecked by a second reviewer (PMF, or EH, or VW) to enhance accuracy and completeness.

The extracted data were then synthesized and analyzed to address the objectives of the rapid review, providing a comprehensive overview of the digital interventions aimed at improving mental well-being and health behavior among university students. Any discrepancies or uncertainties encountered during data extraction were resolved through discussion until consensus was reached among the reviewing team.

### Quality assessment of included studies

The Risk of Bias (RoB) was evaluated using the Cochrane Collaboration’s RoB 2 tool, specifically designed for evaluating randomized controlled trials (RCTs) [38]. This tool assesses the following bias domains: (i) randomization process, (ii) deviations from the intended interventions, (iii) missing outcome data, (iv) outcome measurement, and (v) selection of reported results. Bias ratings were assessed at single outcome level and overall study level. Judgements could be “low” or “high” or express “some concerns”. The overall study rating was: if any single domain was rated as “high risk,” the overall study was classified as “high risk of bias.” If there were some concerns in at least one domain and no domain rated as “high risk,” the study was judged to have “some concerns.” Only studies with all domains rated as “low risk” were given an overall “low risk” rating. Three authors (PMF, EH, and VW) appraised the included studies, and any discrepancies in bias ratings were meticulously addressed through discussion until consensus was reached.

### Data synthesis and analyses

The results are structured in a narrative synthesis outlining the characteristics of the included studies, such as the year of publication, study design, and country of origin. Additionally, the features of the interventions, including the type of DPHI utilized, are described. The synthesis of results is tailored to address the specific research questions stated above, facilitating a comprehensive understanding of the findings.

In determining intervention effectiveness, an intervention is classified as effective if the DPHI shows statistically significant results (*p* < 0.05) when compared to the control group. Conversely, interventions are classified as not effective if no statistically significant differences are observed between the intervention and control groups for the primary outcome.

## Results

### Search outcomes

**Fig. 1:**
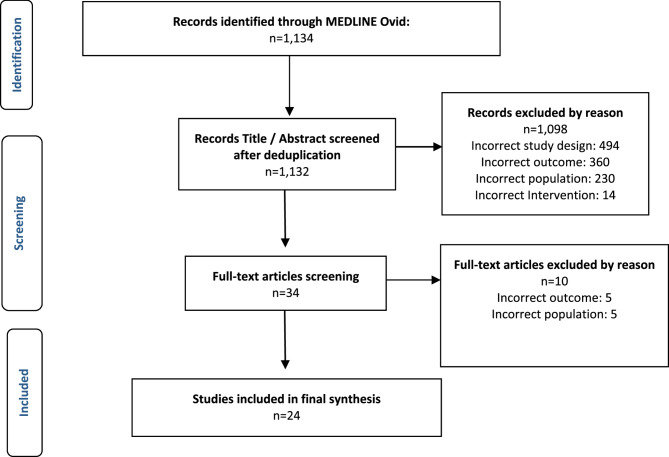
PRISMA Flowchart for literature selection

The electronic database search yielded a total of 1,134 records. Upon title and abstract screening, 1,098 manuscripts were excluded and the most common reasons for excluding articles were that the study did not include the outcomes of interest, the intended population, or study design. Subsequently, 34 articles were included for the full-text review. Following thorough evaluation, 24 articles met the inclusion criteria and were subjected to data synthesis (see Fig. 1).

Detailed information, along with references, for the included studies is provided in Multimedia Appendix 2 (Table 1- Final individual sources of evidence included in the rapid review).

### Characteristics of the included studies

Table 1 (Multimedia Appendix 2) provides a summary of the study characteristics found in the included articles. The studies were published between 2018 and 2023 and were conducted in ten different countries across four continents: Asia, North America, Australia, and Europe. Four studies (16.0%) specifically implemented interventions during the COVID-19 pandemic [39–42]. All 24 included studies adhered to an experimental design. Most of the trials had two intervention arms (72%, *n* = 18), while one included a maximum of eight intervention arms [43]. Six studies (24%) focused on smartphone applications [44–49], while the remaining publications (76%, *n* = 18) examined the effects of different web-based interventions.

The duration of intervention varied from 10 days [39] to 26 weeks [50]. Five studies did not report the duration [45, 51–54]. The overall baseline sample size comprised 8,729 university students, with an average of 349 participants per study, ranging from *n* = 55 [41] to *n* = 2,951 [50]. At study completion or follow-up, a total of 6,037 individuals were analysed, with a mean sample size of 241 participants per study, ranging from *n* = 51 [55] to *n* = 1,095 [45].

The studies covered various health topics, such as improving mental health (68%, *n* = 17), reducing substance use (28%, *n* = 7), enhancing physical activity (36%, *n* = 9), and exploring dietary and eating habits (16%, *n* = 4). The studies aimed to enhance mental wellbeing, encourage healthier lifestyles, and assess the efficacy of different intervention strategies.

### Components and intervention levels in digital mental well-being programs

The interventions included in this review had different components and levels. An overview is presented in Table 1.

**Table 1:** Overview of components and levels of digital interventions by study

Components	Intervention groups/Assignment
Incentive-based approach combined with a smartphone application	Incentivized group and non-incentivized group
Acceptance and Commitment Therapy (ACT)	Randomized assignment to 3 intervention groups (different program delivery methods)
Campus-wide text-messaging program covering various health topics and resources	Campus-wide implementation targeting undergraduate students
Interactive video on Mental Health Literacy (MHL)	Not specified
Depression-focused BITS (Brief Interactive Training Sessions)	Randomized assignment (intervention group or waitlist)
Video conference-based aerobic and resistance training (WeActive) and mindful exercise (WeMindful)	Randomized assignment
Video conference-based aerobic and resistance training (WeActive) and mindful exercise (WeMindful)	Randomized assignment
Internet-Based Cognitive-Behavioral Therapy (CBT) modules	Four single-group longitudinal studies and replication of employee user study
Web-based Mindfulness Virtual Community Intervention (MVC)	Randomized assignment (full-MVC or partial-MVC)
Online multicomponent intervention (ETUCARE)	Randomized assignment (ETUCARE-group or control condition group)
Video conference-based aerobic and resistance training (WeActive) and mindful exercise (WeMindful)	Randomized assignment (WeMindful or WeActive)
Web-based interventions on promoting physical activity (PA) and fruit-vegetable consumption (FVC)	Randomized assignment (PA-first, FVC-first, or control)
Calorie Counting App (MyFitnessPal) - dietary self-monitoring	Randomized assignment (PA-first, FVC-first, or control)
Video conference-based aerobic and resistance training (WeActive) and mindful exercise (WeMindful)	Randomized assignment (WeMindful or WeActive)
Digital Therapeutic Mobile App (BioBase)	Randomized assignment (intervention + waitlist)
Social-media-based support	Quasi-experimental study (experimental group or control group)
Mindfulness Meditation Mobile App (Calm)	Randomized assignment (intervention group, waitlist control group)

A cross-study synthesis revealed that the most common components embedded within DPHIs for the promotion of mental well-being among university students included mindfulness-based strategies (*n* = 6), CBT (*n* = 4), and ACT (*n* = 2). Mindfulness-based interventions were predominantly delivered through mobile applications (e.g., *Calm*,* BioBase*) or virtual community platforms and associated studies generally reported reductions in stress and improvements in resilience and self-compassion [41, 42, 48, 49]. CBT-based interventions were typically delivered via web platforms and ranged in focus from structured mood management programs to interactive psychoeducation modules, with studies reporting mixed outcomes regarding symptoms of depression and anxiety [40, 47, 55]. Physical activity-focused interventions, including ‘*WeActive’* and ‘*WeMindful’*, often used video conferencing to deliver exercise sessions and studies demonstrated positive impacts on physical activity levels, though effects on mental health varied [41, 56]. A small number of interventions used gamification, financial incentives, or multicomponent approaches integrating behavioral and emotional support (e.g., ‘*ETUCARE’* [40]), showing promise but with inconsistent effect depending on user engagement and implementation fidelity.

#### Online interventions

Viskovick et al. [55] introduced *YOLO*, a web-based ACT program, aiming to promote mental health skills and prevent mental health problems. The study employed a random assignment design, allocating participants to one of three intervention groups, exploring different methods for delivering the program. Group 1 followed a structured approach, completing one module per week over a four-week period, with the flexibility to adjust their pace as desired. Group 2 had full autonomy, with four weeks to complete the intervention at their own discretion. Group 3 adopted a sequential approach, accessing a module after completing each previous module, with a mandatory 3-day break between modules.

Queroue et al. [52] took a multimedia approach with their interactive video on mental health literacy, designed to increase awareness and understanding of mental health issues. Through engaging content, they aimed to combat stigma and promote help-seeking behaviors. Meanwhile, Ruehlman and Karoly [57] carried out a study on BITS (Brief Interactive Training Sessions) focused on depression, which aimed at teaching techniques to counteract cognitive distortion and cognitive restructuring. The intervention consisted of short, self-paced sessions with various activities, including watching videos, doing exercises and monitoring progress. Each BITS session addressed specific processes, such as mood control, thought restructuring, mindfulness and PA promotion. The study used random assignment to either an intervention group or a wait-list control group.

The study conducted by Theurel et al. [40] investigated the efficacy of an online multicomponent intervention, *ETUCARE*, designed to improve mental health among university students during the COVID-19 pandemic. The intervention integrated a variety of evidence-based strategies targeting mental health, stress management, procrastination, motivation for learning, sleep, insomnia, self-awareness, emotion regulation, and meaningful relationships. Participants were randomly assigned to either the *ETUCARE* group or a control condition group. Those in the *ETUCARE* group engaged with the online intervention, while the control group completed post-test assessments without participating in the program.

Ahmad et al. [58] conducted a study to assess the efficacy of a Web-Based Mindfulness Virtual Community Intervention (MVC) for improving students’ mental health. The intervention aimed to reduce symptoms of depression, anxiety, and stress, with secondary outcomes including quality of life, life satisfaction, and mindfulness levels. The MVC comprised 12 video-based modules providing psychoeducation on stress management alongside mindfulness practices. Additionally, the intervention included anonymous peer-to-peer discussion forums and group-based, professionally guided live videoconferences. Participants were randomly assigned to either the Full-MVC group, which received all components, or the Partial-MVC group, which lacked live videoconferences.

Physical activity and mindfulness have also been jointly addressed in digital interventions. Marenus et al. [56] explored the effects of video conference-based interventions on PA and mental health outcomes, highlighting the potential of online platforms to facilitate structured exercise and mindful practices. Participants were randomly assigned to two groups: *WeMindful* and *WeActive*. The *WeMindful* group engaged in two 30-minute sessions of yoga and mindful exercise per week for eight weeks, while the *WeActive* group participated in two 30-minute aerobic and resistance training sessions weekly over the same duration. Through randomized assignment, the study aimed to assess the impact of these interventions on both physical and mental health outcomes in a controlled setting. Utilizing the same platform and study design, Friedman et al. [41] investigated the immediate and short-term effects of video conference-based interventions on PA and psychological resilience.

Duan et al. [59] investigated web-based interventions targeting PA and fruit-vegetable consumption (FVC), employing randomized assignment to assess their impact on health-related outcomes. Participants were divided into three groups: PA-first, FVC-first, and a control group. The PA-first group received a 4-week PA intervention followed by a 4-week FVC intervention, while the FVC-first group had the interventions in reverse order. The control group received placebo treatment for 8 weeks.

#### Text messaging

Glowacki et al. [45] introduced *HealthyhornsTXT*, a campus-wide text-messaging program designed to promote positive health behaviors. The intervention involved sending messages covering various health topics, including sleep, stress management, and campus resources related to mental health and well-being. Throughout the semester, process data were collected to assess student engagement with the messages, including tracking replies to text-back keywords and clicks on website links embedded within messages.

#### Apps

Huberty et al. [49] explored the potential of digital interventions for enhancing mental well-being, specifically through the mindfulness meditation mobile app, *Calm*. By employing a randomized assignment design, they investigated the app’s efficacy in reducing stress and fostering mindfulness, self-compassion, and health behaviors. The study by Memon et al. [44] presented an innovative approach to promoting PA and weight loss through the integration of a smartphone application and financial incentives. Divided into incentivized and non-incentivized groups, participants engaged with a smartphone app designed to track PA, with financial rewards based on measured activity levels. Similarly, Hahn et al. [47] explored the effects of the *MyFitnessPal* calorie counting app on health behaviors and mental well-being among undergraduate women. Over approximately one month, the intervention group used the app for dietary self-monitoring, while the control group had no intervention. Using apps as well, Ponzo et al. [48] investigated the *BioBase* app’s effectiveness in reducing stress and improving mental well-being. The app, which included psychoeducational content, mood tracking, and in-the-moment exercises, was assessed through a randomized controlled trial. Nam and Cha [54] focused on the impact of social-media-based support on premenstrual syndrome and PA among female university students. Using the *Fitbit Flex* and a combination of text messaging and email, participants received appraisal, belonging, and self-esteem support over one menstrual cycle. Huberty et al. [49] examined the *Calm* app’s efficacy in reducing stress and enhancing psychological outcomes through an 8-week mindfulness meditation program. Participants in the intervention group had access to guided meditations and tailored programs, while the control group was placed on a waitlist.

### Effectiveness of digital interventions for improving mental well-being

#### Depression, anxiety, and stress reduction

Several studies investigated the impact of various digital interventions on depression, anxiety, and stress symptom reduction, yielding a variety of outcomes. Viskovich et al. [55] reported significant improvements in depression and anxiety symptoms, stress, well-being, self-compassion, and life satisfaction post-intervention across three intervention groups. Similarly, Ahmad et al. [58] observed significant reductions in depression, anxiety, and perceived stress scores, along with improvements in quality of life among participants engaging with mindfulness virtual community interventions.

However, not all interventions showed consistent effects on mental health outcomes. For example, Murray et al. [60] noted marginal effects on anxiety symptoms over time but reported significant reductions in depressive symptoms. Hahn et al. [47] found no impact on secondary mental health outcomes, such as state anxiety, depressive symptoms, or quality of life, in their examination of a dietary self-monitoring app. In contrast, Theurel et al. [40] reported significant reductions in severe psychological distress and anxiety disorder cases among intervention groups. Similarly, Ponzo et al. [48] observed significant reductions in anxiety in the intervention group, with decreases in depression symptoms noted across both intervention and control groups.

Huberty et al. [49] reported a significant reduction in perceived stress among intervention participants. In contrast, Lyzwinski et al. [61] did not find significant differences in stress levels between intervention and control groups. Nevertheless, Duan et al. [59] observed reductions in depressive symptoms among participants engaged in PA and fruit-vegetable consumption interventions.

#### Sleep and stress management

Glowacki et al. [45] conducted a study investigating the impact of a text messaging intervention on health behaviors, focusing primarily on sleep/napping and stress management. The study identified sleep/napping as one of the most relevant messaging topics among participants, with 65% of respondents indicating these topics as relevant. Moreover, the impact of the intervention on behavior change was striking. Specifically, 17 individuals reported that the text messages motivated them to change their sleep/napping behavior.

#### Help-Seeking and depression awareness

Queroue et al. [52] carried out a digital intervention with the aim of reinforcing help-seeking behavior and depression awareness among participants. The intervention group underwent a structured program designed to increase awareness of depression symptoms, reduce stigma around mental health and encourage proactive help-seeking behavior. The results revealed a significant increase in help-seeking behavior and awareness of depression among participants in the intervention compared to the control group.

#### Physical activity and Well-Being

Two studies shed light on the intrinsic relationship between PA and psychological well-being [44, 56]. Marenus et al. [56] investigated the impact of two distinct intervention approaches — *WeActive* and *WeMindful* — on PA levels and psychological well-being. The results suggested encouraging trends, with both intervention groups demonstrating increased PA engagement. However, the outcomes diverged when focusing on psychological well-being. Surprisingly, the *WeActive* group experienced a decrease in psychological well-being scores. Conversely, the *WeMindful* group witnessed an increase in psychological well-being alongside their heightened PA levels. The study underscores the importance of considering not only the quantitative aspects of PA but also its qualitative implications for overall well-being. In contrast, Memon et al. [44] explored the psychological effects of an intervention focused on incentivizing PA through smartphone applications. However, the study did not find significant differences in psychological outcomes between the intervention and control groups.

#### Mindfulness and resilience

Several studies reported an increase in mindfulness scores after the intervention [41, 42, 47–49]. Marenus et al. [42] evaluated the effects of an intervention on mindfulness and resilience. They found that participants had higher mindfulness scores after the intervention, indicating a positive change in their awareness and ability to stay in the present moment. In addition, the intervention had a notable impact on resilience, with participants demonstrating a greater ability to bounce back from adversity. Interestingly, no significant differences were observed between the different intervention groups. Moreover, participants in the intervention group in the study by Ponzo et al. [48] demonstrated significant reductions in stress levels and improvements in mental well-being compared to the waiting list control group. Passive data collected via the *BioBeam* wearable device also showed improved PA patterns.

Friedman et al. [41] focused on resilience as an outcome measure and found a significant increase in resilience scores among participants from pre-test to follow-up in both intervention groups. Furthermore, Hahn et al. [47] reported improved resilience post-intervention in both intervention groups, highlighting the robustness of mindfulness-based approaches in improving psychological resilience across diverse populations.

### Health behavior change embedded in digital mental well-being initiatives

#### Reducing substance use

Seven studies (28%) examined various digital interventions aimed at reducing substance use. Boyle et al. [62] found that the *Spinner* conditions effectively reduced cognitive reactance and alcohol consumption. Riggs et al. [51] investigated the impact of the marijuana *eCHECKUP TO GO* intervention, which was associated with reduced substance use prevalence. This highlights the utility of tailored interventions in targeting specific substance use behaviors and populations. Participants, who received *Theory of Planned Behaviour messages*, as studied by Norman et al. [50], held less favorable views on binge drinking, consumed less alcohol, binged less frequently, and had reduced harmful drinking patterns during their first six months at university.

Fetterling et al. [63] reported several noteworthy findings: The intervention led to increased days since last substance use among frequent cannabis users, indicating its potential effectiveness in reducing consumption frequency. An interaction between sex and descriptive norms influenced the reduction of substance use. Indirect effects were observed for the intervention on substance use through normative perceptions.

Müssener et al. [64] found that both intervention and control groups reported changes in drinking habits due to increased awareness and lifestyle changes. Additionally, participants expressed satisfaction with the intervention structure and message frequency.

Glowacki et al. [45] identified tobacco use (4,7%) and prescription drug misuse (3,8%) as the least relevant message topics, while alcohol use fell in the middle (26,8%). Huberty et al. [49] did not observe significant changes in binge drinking following the intervention.

#### Exploring dietary and eating habits

Regarding dietary and eating habits, five studies (21%) explored changes in dietary and eating habits as a consequence of participation in digital interventions:

- Impact of health messages and social influence

In the study conducted by Glowacki et al. [45], the effectiveness of health messages and social influence on behavior change was examined. Nutrition emerged as one of the most relevant topics with 38% of participants indicating its significance.

- Effects of interventions on dietary behaviors and health outcomes

Duan et al. [59] investigated the effectiveness of web-based interventions in promoting fruit/vegetable consumption, thereby impacting various health outcomes. The study revealed significant improvements in fruit and vegetable consumption post-intervention. Participants exposed to the intervention experienced positive changes in their dietary behaviors. In contrast, Hahn et al. [47] reported no significant changes in eating disorder behaviors or mental health outcomes following their intervention. Despite efforts to promote dietary self-monitoring using a smartphone app, participants did not exhibit substantial improvements in eating behaviors or mental health indicators.

- Secondary outcomes: physical activity and healthy eating

The digital intervention conducted by Huberty et al. [49], investigated changes in participants’ behaviors and outcomes, including healthy eating habits, alongside measures of stress reduction, mindfulness, self-compassion, and feasibility. The results did not indicate a positive effect on healthy eating habits across the intervention groups.

### Risk of bias

**Fig. 2:**
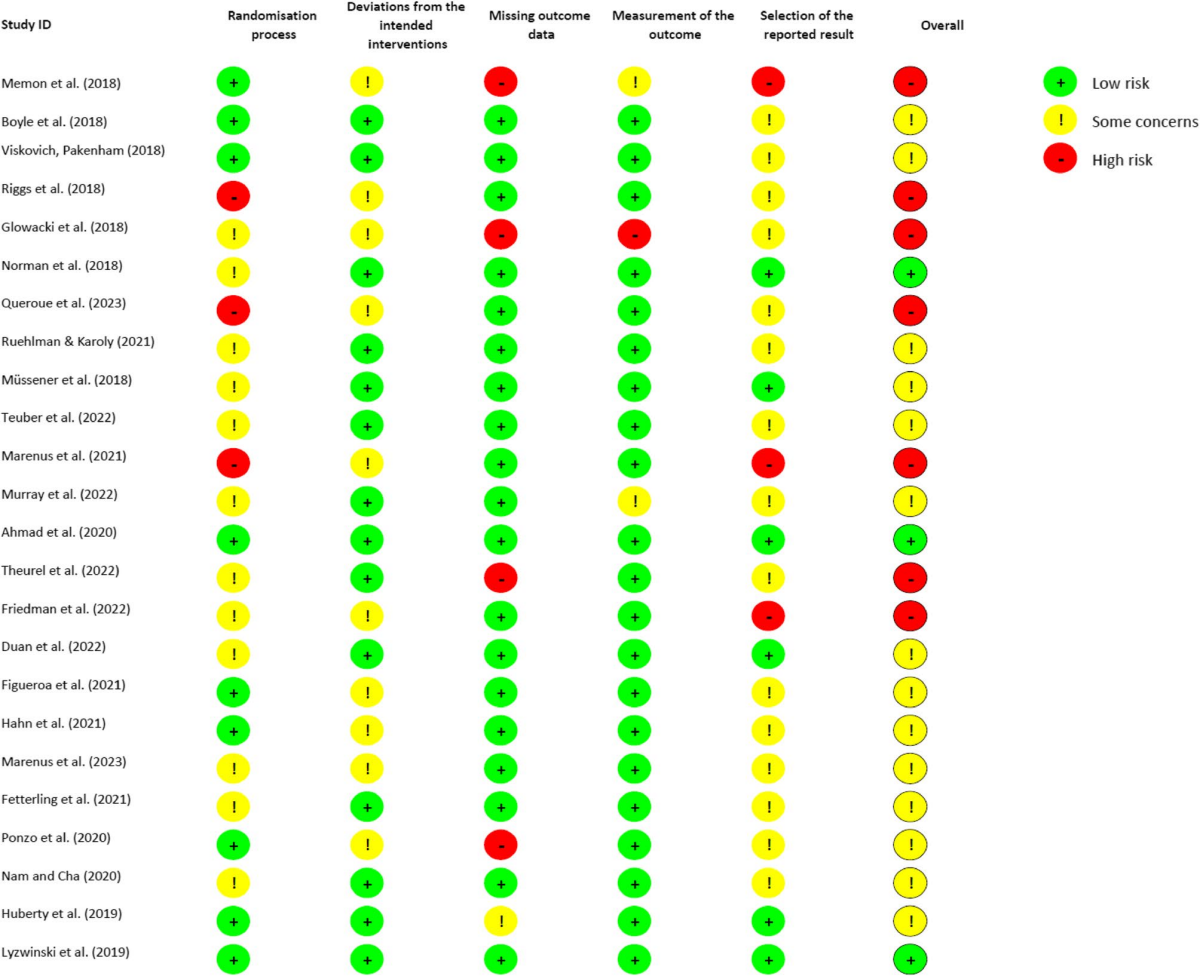
Risk of Bias Assessment of included studies

The overall risk of bias assessment revealed that a significant proportion of studies (58%) were categorized as having “some concerns,” indicating moderate bias across various domains, while 29% were classified as high risk, suggesting considerable bias that could affect the validity of outcomes (see Fig. 2). 13% were at low risk, highlighting overall concerns regarding study quality. Evaluating the randomization process, half of the studies (50%) were rated as having some concerns due to issues, such as inadequate random sequence generation or allocation concealment, with 13% at high risk, suggesting substantial problems, and 38% at low risk, indicating a reasonably robust randomization process. In terms of deviations from intended interventions, none of the studies were classified as high risk, but 42% were rated as having some concerns, while 58% were rated low risk, indicating good adherence to the intended interventions. The assessment of missing outcome data revealed that a majority of studies (79%) were at low risk of bias, 17% were at high risk due to missing data, with only 4% having some concerns in this area. The domain of outcome measurement had the highest percentage of low-risk studies (88%), 8% had some concerns, and 4% were at high risk. Regarding the selection of reported results, a high percentage (63%) of studies were rated as having some concerns, and 13% were at high risk, with 25% at low risk, maintaining transparency and reliability in reporting practices.

## Discussion

Our rapid review provides a comprehensive overview of DPHIs, particularly focusing on their impact on mental well-being and health behaviors among university students. Most interventions utilized web-based platforms (76%), with a smaller proportion relying on smartphone applications (24%). These interventions primarily targeted mental health (68%), substance use reduction (28%), physical activity promotion (36%), and dietary behavior change (16%). In terms of significant effects, 42% of mental health interventions were associated with improvements in the desired direction, while interventions effects for substance use, physical activity, and eating behavior were found in 4%, 25%, and 8% of the studies, respectively. However, a moderate to high risk of bias was observed in a considerable proportion of the studies.

This synthesis highlights the differing effectiveness of intervention types, with psychological outcomes showing greater short-term responsiveness and behavioral outcomes requiring sustained engagement. Key moderating factors—such as intervention duration, user engagement, delivery format, and methodological quality—help explain outcome variability. This review identifies several key themes that synthesize the diverse findings across the included studies.

i) First, digital interventions, such as mobile applications, online counselling, and virtual support communities, emerged as accessible and scalable options for addressing mental health concerns in the university student population. These interventions can offer immediate support and resources, which are particularly valuable in the fast-paced and often stressful university environment. The DPHIs included in this review varied significantly in their approach. However, the DPHIs included in this review varied significantly in their approach, target, and implementation.
ii) Evidence on the effectiveness regarding the DPHIs reveals a nuanced picture that varies by target outcome. Mental health outcomes, including depression, anxiety, stress, well-being, self-compassion, and life satisfaction, showed mixed results [42, 48, 49, 55, 58]. While several interventions reported significant improvements in psychological symptoms—particularly reductions in anxiety, depression, and stress [48, 49, 55, 56, 58]—others failed to demonstrate statistically meaningful changes [47]. This variation suggests that the effectiveness of mental health-focused DPHIs may depend on specific intervention designs and population characteristics, as some approaches appeared more impactful than others in reducing psychological distress.

In contrast, interventions aimed at promoting healthy behaviors (e.g., physical activity, sleep, dietary habits) yielded less consistent results. While certain initiatives targeting physical activity and mindfulness demonstrated improvements in resilience and psychological well-being [41, 42], others showed limited or no significant behavioral change [45, 51, 60, 62, 63]. This may reflect the need for longer intervention durations and sustained engagement to influence lifestyle behaviors meaningfully. When it comes to substance use reduction, DPHIs showed more promising and targeted outcomes. Tailored interventions—such as the *Spinner* model, marijuana *eCHECKUP TO GO* [51, 62], and *Theory of Planned Behavior* messaging [50]—successfully reduced alcohol consumption, binge drinking, and substance use prevalence. These findings suggest that behavior-specific customization and theoretical frameworks can increase DPHI effectiveness in this domain.

These mixed findings across the reviewed studies likely reflect several key moderating factors. First, differences in the type of outcomes assessed—psychological symptoms *versus* behavioral changes—may have influenced how effectiveness was determined and reported in the respective studies. Behavioral outcomes, such as substance use or exercise frequency, often require longer intervention durations and consistent reinforcement to show measurable change, while psychological outcomes may be more immediately responsive to intervention [65, 66]. Second, the follow-up periods varied considerably, which likely contributed to inconsistencies; short-term follow-ups may capture immediate benefits that are not sustained, while longer follow-ups may better assess lasting impact but risk higher attrition [67]. Third, user uptake and engagement emerged as crucial determinants of intervention effectiveness. Studies reporting lower adherence or high dropout rates may underestimate the potential impact of DPHIs, especially when interventions were unguided or lacked personalization [68, 69].The review also highlighted that not all DPHIs are equally effective. Mixed or inconclusive outcomes of some studies suggest that the design, implementation, and context of these interventions play crucial roles in their success. Factors, such as user engagement, personalization of content, and the integration of human support can significantly influence the effectiveness of DPHIs [70]. These findings highlight the complexities of addressing mental health issues through digital means and emphasize the need for a tailored, nuanced approach in the development and deployment of these interventions.

Several interventions - such as BITS, ETUCARE, and various mindfulness-based applications - showed mixed or inconclusive results across studies. These inconsistencies may be explained by several factors. Intervention duration and intensity varied considerably, with some studies implementing short-term or single-session formats, which may not allow sufficient time for measurable behavioral or psychological change. Participant engagement also likely played a role; lower adherence or high dropout rates may have diluted the effects of otherwise well-designed interventions. Additionally, the delivery format (e.g., unguided self-help apps vs. guided virtual sessions) and the degree of personalization may influence both uptake and outcomes. Previous research suggests that user-centered design, interactive features, and the integration of human support, such as coaching or therapist involvement, are associated with higher engagement and better outcomes in digital mental health interventions and better outcomes [71].

iii) Moreover, the review identified weaknesses in research methodology as reflected by the results of the RoB assessment. Many studies had limitations, including small sample sizes, short follow-up periods, and lack of control groups, which affect the reliability and generalizability of their findings [40, 41, 44, 54, 56]. Importantly, studies rated as having a high risk of bias may have influenced the overall interpretation of intervention effectiveness, potentially leading to an overestimation or underestimation of true effects. This heterogeneity in study quality necessitates cautious interpretation of the aggregated findings. To address this in future research, greater emphasis should be placed on methodological rigor—such as conducting well-designed randomized controlled trials that follow standardized reporting guidelines (e.g., CONSORT-EHEALTH) [72], incorporating larger and more diverse samples, and ensuring longer follow-up durations. Additionally, exploring the long-term effects of digital interventions is essential for understanding their sustained impact on mental well-being and health.

This review also highlights the role of DPHIs in promoting help-seeking behavior and increasing depression awareness. Digital interventions can effectively empower individuals to recognize the signs of depression and take proactive steps to seek support and assistance when needed [44]. By fostering a greater understanding of mental health issues and promoting a proactive approach to seeking help, interventions, such as the one conducted by Queroue et al. [52], have the potential to contribute to early detection and intervention of depression, ultimately improving outcomes for individuals experiencing mental health challenges. This underscores the importance of implementing evidence-based interventions that do not only address the symptoms of depression, but also the barriers to seeking help, thereby promoting a supportive environment for mental health awareness and well-being.

Another important factor explored in this rapid review was the integration of health behavior interventions into digital mental wellbeing interventions. These DPHIs have shown particular promise in reducing substance use through personalized approaches. Their effectiveness in targeting specific health behaviors has been highlighted by studies that offer personalized support and resources that can be easily accessed by individuals [51, 62]. Using technology, these interventions can provide continuous, real-time assistance and monitoring, which makes them a powerful tool for promoting healthier lifestyles and improving overall mental wellbeing [73].

Recently, there has been a growing interest in exploring interventions aimed at enhancing mindfulness and resilience, both crucial components of mental well-being. Several studies investigated the effectiveness of various interventions in achieving these goals, yielding valuable insights into their impact and efficacy [42, 48, 49, 61]. These interventions demonstrated considerable potential in fostering healthier lifestyles and improving overall quality of life. As technology continues to evolve, further research and innovation in this field hold the promise of revolutionizing public health practices and reaching broader populations with tailored, evidence-based interventions.

Several limitations of our rapid review must be acknowledged. In a rapid review, time allocated for comprehensive literature searching, screening, and data extraction is limited. This constraint may have resulted in the omission of relevant studies or the inclusion of less rigorous evidence. The search strategy employed in this rapid review was based on only one single database (OVID MEDLINE) and, consequently, this may have led to a less exhaustive retrieval of studies, several important articles that could have influenced the results may have been left out in the process. Although we have assessed the quality of the included studies, the rapid review process sometimes allows for a more superficial quality assessment compared to traditional systematic reviews. This may have affected the robustness of the conclusions obtained.

The results of this rapid review may have limited generalizability due to the specific context and population studied (university students). Extrapolation of the results to different contexts or wider populations should be done with caution. Furthermore, the included studies demonstrated considerable heterogeneity in terms of intervention design, delivery modalities, target outcomes, and measurement tools. This diversity limited the possibility of directly comparing results across studies. As a result, our synthesis focuses on identifying overarching trends and patterns rather than establishing quantitative estimates of intervention efficacy. This heterogeneity also impacts on generalizability of our findings and should be considered when interpreting the results.

In conclusion, although DPHIs hold substantial promise for improving mental well-being and promoting healthy behaviors among university students, their varied effectiveness calls for more rigorous research. Based on the findings of our review, universities should consider integrating DPHIs into their existing mental health services as a complementary resource. Given the increasing demand for mental health support and the limitations of traditional counseling services, DPHIs can offer students immediate and scalable support, easing the burden on mental health professionals, while expanding access to care.

To improve the effectiveness of these interventions, it is essential to prioritize personalization and user engagement. Digital tools should be designed to adapt to individual needs and preferences, incorporating features, such as real-time feedback and tracking of individual progress. Engaging students with intuitive and interactive platforms that offer personalized content is key to ensuring high levels of participation. Additionally, combining digital interventions with human support, such as online counseling or peer mentoring, can further increase engagement and effectiveness.

Collaboration between mental health professionals and technology developers is another critical element. Universities and health organizations should work closely with tech developers to ensure that DPHIs are evidence-based, clinically sound, and appropriately tailored to the needs of students. Such partnerships help create digital solutions that do not only meet the demands of students but also align with best practices in mental health care.

Accessibility is another important consideration. Universities and health providers must ensure that DPHIs are available to all students, including those from underserved or marginalized communities. This includes providing resources to address barriers, such as limited access to technology, lack of internet connectivity, or technological literacy. Offering subsidized devices, multilingual options, or training on how to use digital platforms effectively can help ensure that DPHIs are inclusive and accessible to every student.

Moreover, universities and public health organizations should establish clear mechanisms to assess the effectiveness of DPHIs. Gathering user feedback, tracking engagement metrics, and conducting both short-term and long-term evaluations can provide valuable insights for refining these interventions. Pilot testing DPHIs before a wide-scale implementation will also help identify potential barriers to success which can be addressed while planning for implementation.

Public health policymakers can play a significant role by supporting research on DPHIs and prioritizing funding for studies that assess their long-term effectiveness. Policymakers should focus on creating an environment that encourages innovation while ensuring that digital interventions adhere to regulatory standards and are based on scientific evidence. By fostering research and the development of high-quality DPHIs, they can contribute to improving overall student health.

Through these collective efforts, universities, mental health providers, and policymakers can better support the mental well-being of students and broader communities, ensuring that digital health interventions are effective, equitable, and sustainable.

## Supplementary Information

Supplementary Material 1.

Supplementary Material 2.

Supplementary Material 3.

## Data Availability

All data generated or analysed during this study are included in this published and its supple-mentary information files.

## Abbreviations

WHO: World Health Organization
DPHIs: Digital Public Health Interventions
APPs: Applications
RoB: Risk of Bias
ACT: Commitment Therapy
BITS: Brief Interactive Training Sessions
MVC: Mindfulness Virtual Community Intervention
PA: Physical Activity
FVC: Fruit-Vegetable Consumption
