# Supplementary material for: Digital public health interventions for the promotion of mental well-being and health behaviors among university students: a rapid review
Source: BMC Public Health. 2025 Jul 18;25:2500. doi: 10.1186/s12889-025-23669-1 (PMC12273279; doi:10.1186/s12889-025-23669-1)
Supplement: Supplementary file 1 — Supplementary Material 1. [file 12889_2025_23669_MOESM1_ESM.docx]

**OVID MEDLINE:**

Search Strategy:

| **#** | **Searches** | **Results** |
| --- | --- | --- |
| 1 | Universities/ | 52458 |
| 2 | (universit* or college*).mp. | 615924 |
| 3 | ((higher education or tertiary education or undergraduate* or graduate or postgraduate* or bachelor* or master* or “state examination”) adj5 (campus* student* or setting* or population or institution*)).mp. | 4860 |
| 4 | 1 or 2 or 3 | 618391 |
| 5 | physical activit*.mp. | 153158 |
| 6 | Exercise/ | 141760 |
| 7 | (alcohol* adj5 (intake* or consum* or behavio* or use* or misuse* or abus* or risk* or disorder* or depend* or binge drink*)).mp. | 180859 |
| 8 | Cigarette Smoking/ or Smoking/ | 152270 |
| 9 | "Tobacco Use"/ | 3567 |
| 10 | ((smok* or tobacco or cigar* or vaporize* or e-cigar*) adj5 (status or use* or behavio*)).mp. | 115185 |
| 11 | Alcohol Drinking/ | 75368 |
| 12 | ((drug* or substance* or cannabis or marijuana or weed, hash) adj5 (use* or abuse* or misuse* or depend* or disorder*)).mp. | 1281156 |
| 13 | ((health or healthy or lifestyle) adj3 (behavio* or factor*)).mp. | 169843 |
| 14 | 5 or 6 or 7 or 8 or 9 or 10 or 11 or 12 or 13 | 1945523 |
| 15 | Mental Health/ | 61659 |
| 16 | Self Concept/ | 60855 |
| 17 | "Quality of Life"/ | 269256 |
| 18 | Personal Satisfaction/ | 24336 |
| 19 | Happiness/ | 5501 |
| 20 | Resilience, Psychological/ | 8370 |
| 21 | Depression/ or Stress, Psychological/ | 269609 |
| 22 | Anxiety/ | 106846 |
| 23 | ((psychological or mental) adj5 (wellbeing or well being or health or illness* or disorder* or MD* or “Mood disorder*” or “Affective disorder*”)).mp. | 502100 |
| 24 | (wellness or self concept or self esteem or self worth or self-confidence or "quality of life" or satisfaction or happiness or affect or resilience or flourishing or stress or "psychological distress" or depression or depress* or anxiety).mp. | 3107548 |
| 25 | 15 or16 or 17 or 18 or 19 or 20 or 21 or 22 or 23 or 24 | 3398339 |
| 26 | (online based* or mobile* or ehealth* or “electronic mental health” or "e-mental health" or online-based* or internet-based* or web-based* or computer-based).mp. | 210902 |
| 27 | (digital intervent* or online intervent* or internet intervent* or internet bas* intervent* or web intervent* or web-based intervent* or online treatment or computer bas* intervent* or computer intervent* or computer assisted intervent* or e-health or m-health or smartphone* or electronic* or mobile applications or public health or population-level).mp. | 902884 |
| 28 | 26 or 27 | 1073715 |
| 29 | 4 and 14 and 25 and 28 | 1839 |
| 30 | **limit 29 to (english language and yr="2018 -Current")** | 1134 |
