# Supplementary material for: Digital public health interventions for the promotion of mental well-being and health behaviors among university students: a rapid review
Source: BMC Public Health. 2025 Jul 18;25:2500. doi: 10.1186/s12889-025-23669-1 (PMC12273279; doi:10.1186/s12889-025-23669-1)
Supplement: Supplementary file 2 — Supplementary Material 2. [file 12889_2025_23669_MOESM2_ESM.docx]

| Author Year  Country of origin | Titel | Primary aim | n | Study participants  age: range (mean) | Type of Intervention (Program) | Intervention duration (weeks) | Intervention arms (n) | Outcomes |
| --- | --- | --- | --- | --- | --- | --- | --- | --- |
| Memon et al. (2018) Pakistan | The effectiveness of an incentivized physical activity programme (Active Student) among female medical students in Pakistan: A Randomized Controlled Trial | Determine the efficacy of an incentive-based approach combined with a smartphone application in promoting physical activity and weight-loss among female medical students | 56 | 18-25 (20.63) | Randomised controlled trial | 5 | 2 | Within-Group Weight Loss at the end of the intervention: significant for both groups (p<0.05) |
| Boyle et al. (2018)  USA | Increasing Chance-Based Uncertainty Reduces Heavy Drinkers' Cognitive Reactance to Web-Based Personalized Normative Feedback | Examine the potential of incorporating chance-based uncertainty (= popular game mechanic linked to motivation + attention in educational digital games) to mitigate psychological reactance in heavy-drinking college students receiving web-based Personalized Normative Feedback | T1: 141 T2: 138 T3: 138 | n. r. | Randomised controlled trial | 3 | 4 | Drinking Norms: ø significant effect for condition  Cognitive Reactance: Significant differences  Alcohol Consumption:Significant differences. Better reduced drinking than others |
| Viskovich, Pakenham (2018) Australia | Pilot evaluation of a web-based acceptance and commitment therapy program to promote mental health skills in university students | Evaluate the YOLO web-based ACT (Acceptance and Commitment Therapy) program for university students over four weeks, incorporating all six ACT processes. Excludes face-to-face contact and incentives. Investigate three delivery modes to minimize attrition. | T1: 130 T2: 51 | 18-62 (26.34) T1T2: 18-62 (27.06) PP: 16-62 (28.28) | Quasi-experimental study | 4 | 3 | Significant improvements from pre- to postintervention in depression, anxiety, stress, well-being, self-compassion, life satisfaction. |
| Riggs et al. (2018)USA | Marijuana eCHECKUPTO GO: Effects of a personalized feedback plus protective behavioral strategies intervention for heavy marijuana-using college students | Test the direct and moderated program effects of an adapted version of the Marijuana eCHECKUPTO GO, a web-based marijuana use intervention providing university-specific personalized feedback with normative information and PBS to students attending a university in a state with legalized adult recreational marijuana. | T0:298  Analysed: 227 | n. r. (19.97) | Randomised controlled trial | 12 | 2 | Marijuana eCHECKUP TO GO participants reported reduced use prevalence compared to HSM comparison participants at 6-week posttest |
| Glowacki et al. (2018) USA | HealthyhornsTXT: A Text-Messaging Program to Promote College Student Health and Wellness | Examine the feasibility and acceptability of a campus-wide, health text–messaging program. | 1095 | 18-24 (n. r.) | HealthyhornsTXT - a campus wide text-messaging program | Data collection: approx. 21.65 (08/2015 - 12/2015) | 1 | Sleep/napping (65%), stress management (61%), nutrition (38%); body image (19%), tobacco use (5%), prescription drug misuse (4%) |
| Norman et al. (2018) United Kingdom | A randomized controlled trial of a brief online intervention to reduce alcohol consumption in new university students: Combining self-affirmation, theory of planned behaviour messages, and implementation intentions | Test whether combining (1) messages that target key beliefs from the theory of planned behaviour (TPB) that underlie binge drinking, (2) a self-affirmation manipulation to reduce defensive processing, and (3) implementation intentions (if-then plans to avoid binge drinking) reduces alcohol consumption in the first 6 months at university. | T0 :2951 Final Sample: 657 | T0: n. r. (18.76) | Randomised controlled trial | 26 | 8 | Binge Drinking Frequency: Time (p<0.001): Peak at 1st week, declined, remained stable at 1 & 6 months Message condition (p=0.04): Decrease in viewers  significant effect on all cognitions, all increased significantly over time effects via self-efficacy |
| Queroue et al. (2023)France | An interactive video increasing French students’ mental health literacy: a mixed-methods randomized controlled pilot study | Evaluate the appreciation and effectiveness of an interactive video on French University students’ MHL (knowledge about depression and suicidal behavior, mental health help-seeking behaviors, stigma and misconceptions about mental health). | 101 | Age: median contr. 20.0; and interv. 21.0 | Randomised controlled trial | n. r. | 2 | GHSQ Score Correlation with learning and behavioral changes |
| Ruehlman & Karoly (2021) USA | A pilot test of Internet-delivered brief interactive training sessions for depression: Evaluating dropout, uptake, adherence, and outcome | To improve the uptake, adherence, and dropout rates of Internet-based programs for depression by introducing Brief Interactive Training Sessions (BITS). | T0: 68 Analysed: 52 | n. r. (19.40)* | Randomised controlled trial | 7 | 2 | Through a mixed-methods approach, semi- structured interviews were also conducted with the intervention group to collect information on the appreciation of the interactive video |
| Müssener et al. (2018) Sweden | A Text Message–Based Intervention Targeting Alcohol Consumption Among University Students: User Satisfaction and Acceptability Study | Evaluate self-reported changes in drinking habits in the intervention and control groups, and assess user satisfaction in the intervention group. Explore the experience of being allocated to the control group. | T0 (allocated): 896 Follow-up: 815 | n.r. 24 ; r. 24 | Randomised controlled trial | 6 | 2 | Changes in Drinking Habits and Reasons for Reduced Consumption |
| Teuber et al. (2022) Germany | Nudging digital physical activity breaks for home studying of university students—A randomized controlled trial during the COVID-19 pandemic with daily activity measures | Evaluate the impact of a 10-day digital nudging intervention on encouraging physical activity (PA) breaks among university students during the COVID-19 pandemic. | T0: 81 Final Sample: 57 | 18-32 (23.52*) | digital PA break offer “Bewegungssnack digital” [in English “exercise snackdigital” (ESD) | 1.43 (10 days) | 2 | Only daily home study hours had a significant influence |
| Marenus et al. (2021)USA | Feasibility and Effectiveness of the Web-Based WeActive and WeMindful Interventions on Physical Activity and Psychological Well-Being | Examining the feasibility and effectiveness of aerobic and resistance training (WeActive) and mindful exercise (WeMindful) interventions in improving physical activity, psychological well-being and subjective vitality among college students. | 77 | n. r. (23.43) | aerobic and resistance training (WeActive)mindful exercise (WeMindful) | 8 | 2 | Both groups observed increased participants in "high" and "moderate" activity Psychological Well-Being |
| Murray et al. (2022) USA | The Impact of Web-Based Physical Activity Interventions on Depression and Anxiety Among College Students: Randomized Experimental Trial | This study aims to determine the effects of a web-based aerobic resistance exercise intervention (WeActive) and a web-based yoga mindfulness exercise intervention (WeMindful) on depression and anxiety symptoms in college students. | T0: 77 Follow-up: 77 Analysis: 74 | n. r. (23.51*) WeActive: 23.02 WeMindful (24.31) | web-based aerobic resistance exercise intervention (WeActive) and a web-based yoga mindfulness intervention (WeMindful) | 8 | 2 | Both groups show no or doubtful depression levels at T0 Anxiety Scores  WeActive:WeMindful mild anxiety range, Health, Exercise, Yoga History |
| Ahmad et al. (2020) Canada | An Eight-Week, Web-Based Mindfulness Virtual Community Intervention for Students’ Mental Health: Randomized Controlled Trial | This study investigated the efficacy of the Web-based Mindfulness Virtual Community (MVC) intervention in reducing symptoms of depression, anxiety, and stress among undergraduate students in Toronto, Canada. The secondary outcomes included quality of life, life satisfaction, and mindfulness. | T1: 113 T2: 106 T3: 109 | T1: n. r. (24.8) T2: n. r.  T3: n. r. | Web-based Mindfulness Virtual Community (MVC) | 8 | 4 | Significant reductions for F-MVC  Anxiety Score: significant reduction  Quality of Life: significant increase for F-MVC Student Life Satisfaction: significant increase in score for F-MVC  Mindfulness Level : significant improvement for F-MVC |
| Theurel et al. (2022)France | Promoting University Students’ Mental Health through an Online Multicomponent Intervention during the COVID-19 Pandemic | To address the pressing issue of mental health among French university students during the COVID-19 pandemic. It specifically focuses on evaluating the effectiveness of the 8-week online intervention, ETUCARE, in improving psychological well-being and reducing symptoms of distress, anxiety, and alcohol consumption. | T0 (pre-test): 103T1 (final sample): 58 | IG: 20.3, 18-25CG: 19.8, 18-23 | ETUCARE (multitheoretical online self-help program for improving university students’ mental health) | 8 | 2 | Well-being Scores: significant increase Depression Scores: marginally significant decrease Significant reduction in clinical cases of severe psychological distress |
| Friedman et al. (2022) USA | Enhancing Physical Activity and Psychological Well-Being in College Students during COVID-19 through WeActive and WeMindful Interventions | Examine the immediate and short-term effects of aerobic and resistance training (WeActive) and mindful exercise (WeMindful) virtual interventions in improving physical activity (PA) and resilience among college students | 55 | n. r.  Exclusion criteria: <18 years old | WeActive & WeMindful | 8 | 2 | Total Physical Activity: Significant main effect of time  WeActive and WeMindful: significantly increased resilience scores |
| Duan et al. (2022) China | The Effectiveness of Sequentially Delivered Web-Based Interventions on Promoting Physical Activity and Fruit-Vegetable Consumption Among Chinese College Students: Mixed Methods Study | To examine the effectiveness of 2 sequentially delivered 8-week web-based interventions on physical activity, fruit-vegetable consumption (FVC), and health-related outcomes (BMI, depression, and quality of life) and the differences in the intervention effects between the 2 sequential delivery patterns. | 552 (intention-to-treat) | 18-24 (19.99) | PA-first module + FVC-first module | 8 | 3 | Intervention Effects on weekly PA and daily servings of FVC: Significant changes in both over time fpr FVC-first) |
| Figueroa et al. (2021)USA | Daily Motivational Text Messages to Promote Physical Activity in University Students: Results From a Microrandomized Trial | Assessed the effects of sending daily motivational and feedback text messages in a microrandomized clinical trial (MRT) on changes in physical activity from one day to the next in a student population | Basline: n = 93T1: n = 82 | Basline: n. r. (20.2) | mHealth app "DIAMANTE" and text-messaging platform HealthySMS | 6 | 2 | Overall Impact: daily step change: today’s–yesterday’s step count  Psychological Questionnaires- significantly increased from T0 to follow-up |
| Hahn et al. (2021) USA | Introducing Dietary Self-Monitoring to Undergraduate Women via a Calorie Counting App Has No Effect on Mental Health or Health Behaviors: Results From a Randomized Controlled Trial | To determine if introducing dietary self-monitoring via a popular smartphone app to undergraduate women impacts eating disorder risk, other aspects of mental health, or health behaviors including dietary intake and physical activity. | 200 (100 / 100) | n. r. (20.2) | MyFitnessPal | 1 month (appprox. 4 weeks) | 2 | - no impact on secondary mental health outcomes: state anxiety, depressive symptoms, body image, quality of life  - no influence on dietary intake, physical activity, social media use, or screen time - no differentially change from BMI by intervention condition - no association between intervention condition and forms of physical activity self-monitoring |
| Marenus et al. (2023) USA | Web-Based Physical Activity Interventions to Promote Resilience and Mindfulness Amid the COVID-19 Pandemic: A Pilot Study | Examine the effectiveness of two interventions, WeActive and WeMindful, in improving resilience and mindfulness among college students during the unique challenges posed by the COVID-19 pandemic. | 72 | - | WeActive and WeMindful | 8 | 2 | Resilience Mean: slightly below the US population  Mindfulness Mean: moderate mindfulness Significant main effect of Intervention on Resilience and Mindfulness |
| Fetterling et al. (2021) USA | Moderated Mediation of the eCHECKUP TO GO College Student Cannabis Use Intervention | Investigate the impact and mechanisms of the Marijuana eCHECKUP TO GO (eCTG) program on near-daily cannabis users among college students. | Baseline 227 (I: 144 / C: 154) | n. r. (19.97) | eCHECKUP TO GO (eCTG) program | 6 | 2 | Increase in days since last use among frquent cannabis users |
| Ponzo et al. (2020)United Kingdom | Efficacy of the Digital Therapeutic Mobile App BioBase to Reduce Stress and Improve Mental Well-Being Among University Students: Randomized Controlled Trial | To test the efficacy and sustained effects of using a mobile app (BioBase) and paired wearable device (BioBeam), compared with a waitlist control group, on anxiety and well-being in university students with elevated levels of anxiety and stress. | T0: 146T1: 129T2: 116T3 (follow up): 123 | T0: n. r. (19.88*)T1: 18-25 (19.9*)T2: 18-25 (19.92*)T3: 18-25 (19.96*) | BioBase and BioBeam | 4 | 2 | Anxiety: Significant main effect  Mental Well-Being: significant main effect  Depression: decreased depressive symptoms |
| Nam and Cha (2020) South Korea | Effects of a social-media-based support on premenstrual syndrome and physical activity among female university students in South Korea | Investigate the effects of social-media-based support on premenstrual syndrome (PMS) and physical activity among female South Korean university students. | T0: 68 (34 / 34)  T1 (follow-up): 64 | T0: n. r.  T1: n. r. (21.96*) | Fitbit Flex | n. r. | 2 | ø statistical differences in the remaining four premenstrual symptom scores: endogenous depressive features, hysteroid features, increased well-being, and miscellaneous mood/behavior changes Physical Activity: significant increase in overall physical activity level per week |
| Huberty et al. (2019)USA | Efficacy of the Mindfulness Meditation Mobile App “Calm” to Reduce Stress Among College Students: Randomized Controlled Trial | Test the initial efficacy and sustained effects of an 8-week mindfulness meditation mobile app—Calm—compared to a wait-list control on stress, mindfulness, and self-compassion in college students with elevated stress - Explore the intervention’s effect on health behaviors and the feasibility and acceptability of the app | T0: 88T1 (post intervention)T2 (follow-up): 72 | T0: n. r. (21.18*)T1: n. r. T2: n. r. | Calm | 8 | 2 | Perceived Stress: significant reduction; Mindfulness: significant improvements in total mindfulness; Self-Compassion: significant improvements; Sleep Quality: significant decrease in sleep disturbance  Binge Drinking: ø significant  Physical Activity Participation: ø significant changes  Healthy Eating: ø significant changes |
| Lyzwinski et al. (2019) Australia | The Mindfulness App Trial for Weight, Weight-Related Behaviors, and Stress in University Students: Randomized Controlled Trial | Test the effectiveness, acceptability, and feasibility of a student-tailored mindfulness app for weight, weight-related behaviors, and stress. | T0: 90 T1: (follow-up): 72 (36 / 36) | T0: 18-24 (20.19) T1: n. r. | My Student Mindfulness App | 11 | 2 | Weight Changes: no significant  Mindfulness: statistically significant differences Mindfulness levels; Eating: statistically significant differences  Emotional Eating: Significant difference Uncontrolled Eating: Significant difference Cognitive Restraint: no statistically significant ; Stress: no significant difference; Physical Acticity: higher MET moderate activity levels ; Physical Activity Level Categories: no significant differences |
